# Adrenal hematopoiesis in a pediatric patient with spherocytosis: A case report and literature review

**DOI:** 10.1016/j.radcr.2025.04.039

**Published:** 2025-05-07

**Authors:** Rawa Bapir, Ismaeel Aghaways, Sharaza Qadir Omer, Rezheen J. Rashid, Shaho F. Ahmed, Hadeel Adnan Yasseen, Hardi M. Dhahir, Honar O. Kareem, Dilan S. Hiwa, Berun A. Abdalla, Fahmi H. Kakamad

**Affiliations:** a Smart Health Tower, Madam Mitterrand Street, Sulaymaniyah, Kurdistan, Iraq; b Department of Urology, Sulaymaniyah Teaching Hospital, Sulaymaniyah, Kurdistan, Iraq; c Kscien Organization for Scientific Research (Middle East Office), Sulaymaniyah, Kurdistan, Iraq; d College of Medicine, University of Sulaimani, Sulaymaniyah, Kurdistan, Iraq; e Radiology Department, Hiwa Hospital, Sulaymaniyah, Kurdistan, Iraq

**Keywords:** Extramedullary hematopoiesis, Incidentaloma, Erythropoiesis, Adrenal gland, Spherocytosis

## Abstract

Extramedullary hematopoiesis (EMH) is extremely rare in the adrenal glands, especially in children. Because this is an underrecognized condition, the vast majority of cases are diagnosed only after adrenalectomy. An 11-year-old female with hereditary spherocytosis presented with dull right hypochondrial pain. Ultrasonography showed chronic calculous cholecystitis, and splenomegaly. A computed tomography revealed a 4 × 1.7 cm mass in the right adrenal gland with no fat content. Laparoscopic cholecystectomy with adrenalectomy was done. Histopathological examination of the adrenal mass revealed hematopoietic precursors demonstrating megakaryocytes, admixed myeloid precursors, and erythroid precursors without adipose tissue. Nearly 30 cases of adrenal EMH have been documented in the literature. Despite being a benign condition and most of the cases diagnosed incidentally, adrenalectomy was done for the majority of patients even though conservative treatment has shown good results in several case reports. Approximately 75% of cases occurred in the right adrenal gland, with bilateral and left side constituting 12.5% each. Patients with hereditary spherocytosis may present with an adrenal mass due to EMH, even in pediatric age groups. In patients with known hematologic diseases, preoperative diagnosis through functional imaging or biopsy may prevent the need for surgical intervention.

## Background

Hematopoiesis is an intricate physiological process that generates and differentiates blood cellular components, including red blood cells, white blood cells, and platelets, from rare undifferentiated multipotent hematopoietic stem cells, which are generated solely during the embryonic development of a person’s life. This process occurs in a fetus's yolk sac, spleen, and liver while the bone marrow is still being developed. When hematopoiesis occurs in any location other than the bone marrow, it is termed extramedullary hematopoiesis (EMH). This compensatory response ensures adequate blood cell production when the bone marrow cannot meet the body's demands. For instance, in cases of leukemia, myelofibrosis, and lymphoma, the normal function of the bone marrow is disrupted. In addition to increased red blood cell turnover, as in cases of sickle cell disease and thalassemia. In the scenarios above, the EMH most commonly occurs in the spleen, liver, or lymph nodes [[1], [2], [3]].

Hereditary spherocytosis is a prevalent genetic condition marked by anemia, jaundice, and an enlarged spleen. This disorder is observed globally and is particularly frequent among individuals of northern European descent, making it the most widespread inherited anemia in this population. The clinical manifestations vary significantly, with many patients experiencing well-managed hemolytic anemia, while others may be asymptomatic or suffer from severe hemolytic anemia necessitating red blood cell transfusions. The fundamental issue in hereditary spherocytosis is the reduction in membrane surface area, which leads to decreased flexibility of the red blood cells. This is due to defects in membrane proteins such as ankyrin, α spectrin, β spectrin, band 3, or protein 4.2. Numerous isolated mutations have been identified in the genes coding for these proteins. Abnormal spherocytes are sequestered and destroyed in the spleen, which is the primary cause of hemolysis in this condition. Frequent complications include gallstones, hemolytic episodes, and aplastic crises [4].

The adrenal glands are 2 small endocrine glands positioned atop the kidneys in the retroperitoneum. Each gland consists of 2 separate parts, both functionally and embryologically: the medulla, which is responsible for synthesizing catecholamines, and the cortex, which is vital for sustaining life and produces 3 steroid hormones [5].

Nearly 30 cases of adrenal EMH have been documented in the genuine scientific literature [2,3,[6], [7], [8], [9], [10], [11], [12], [13], [14], [15], [16], [17], [18], [19], [20], [21], [22], [23], [24], [25], [26], [27], [28], [29], [30]]. However, the current study aims to present a pediatric patient with hereditary spherocytosis with EMH as an adrenal mass leading to surgical resection of the mass. This case report was written per the CaReL guidelines [31].

## Case presentation

### Patient information

An 11-year-old female patient presented with the only complaint of dull right hypochondrial pain with no radiation to other sites. She had no urinary symptoms. She was a known case of hereditary spherocytosis diagnosed 45 days after birth. The patient received 7 blood transfusions until the age of 4 years. The patient was on folic acid supplementation. The past surgical history of the patient was negative, and her family history was negative for any blood disorders as well.

### Physical examination

The patient's abdomen was soft and flat, with no tenderness or palpable masses. There was no significant physical exam finding.

### Diagnostic approach

**Fig. 1:**
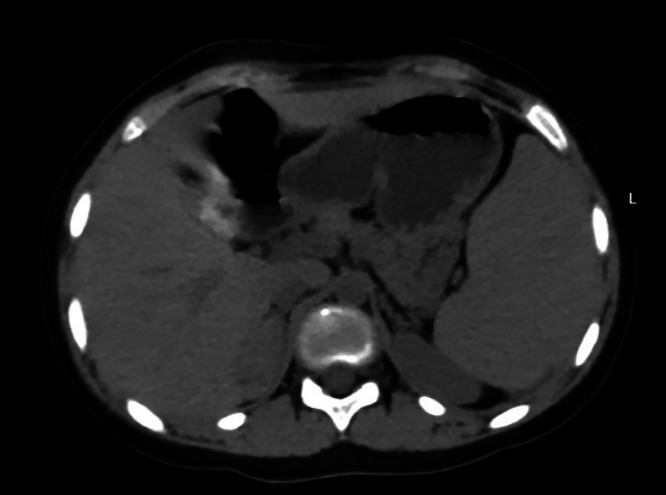
precontrast CT scan of the right adrenal soft tissue lesion (48HU), no fat or calcific content.

**Fig. 2:**
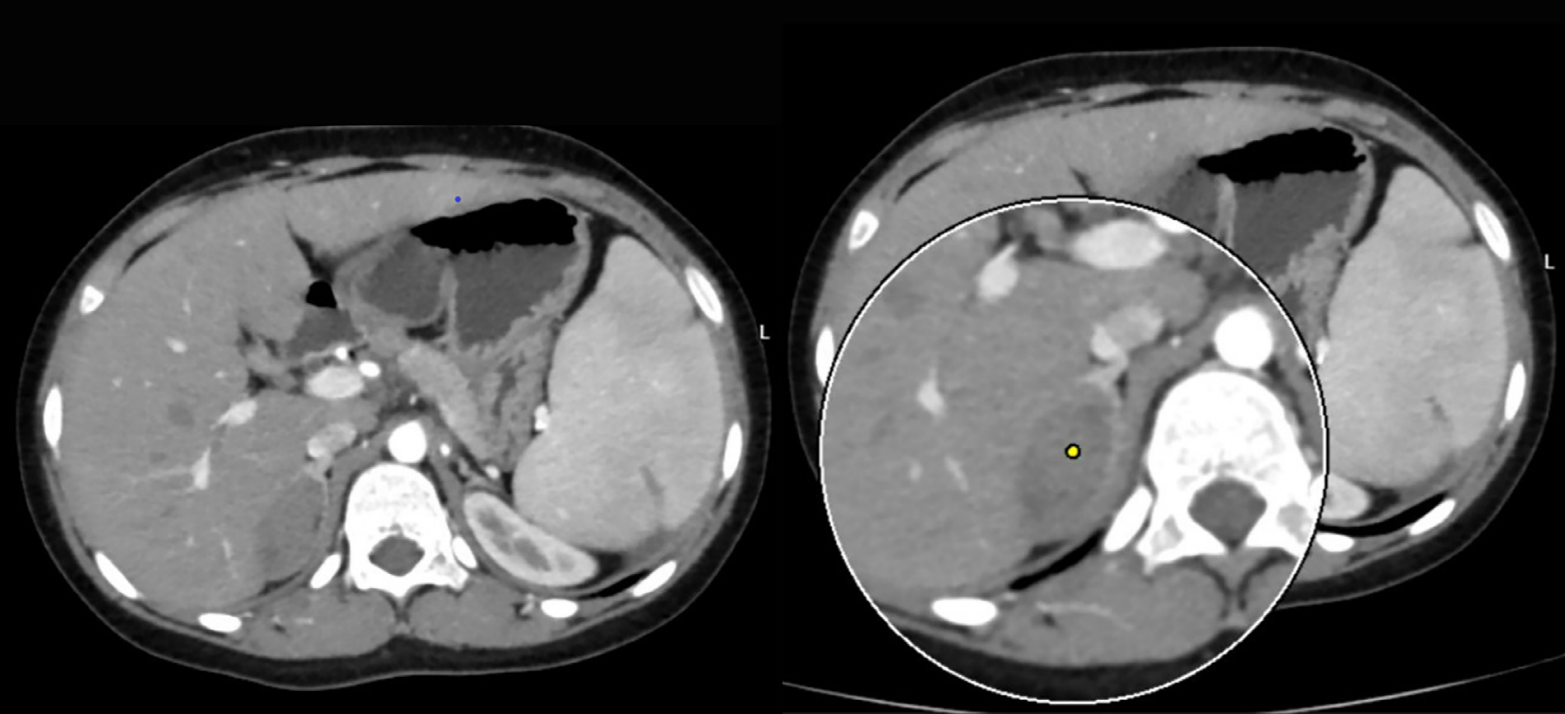
The arterial phase showing right adrenal lesion with mild enhancement (61HU).

**Fig. 3:**
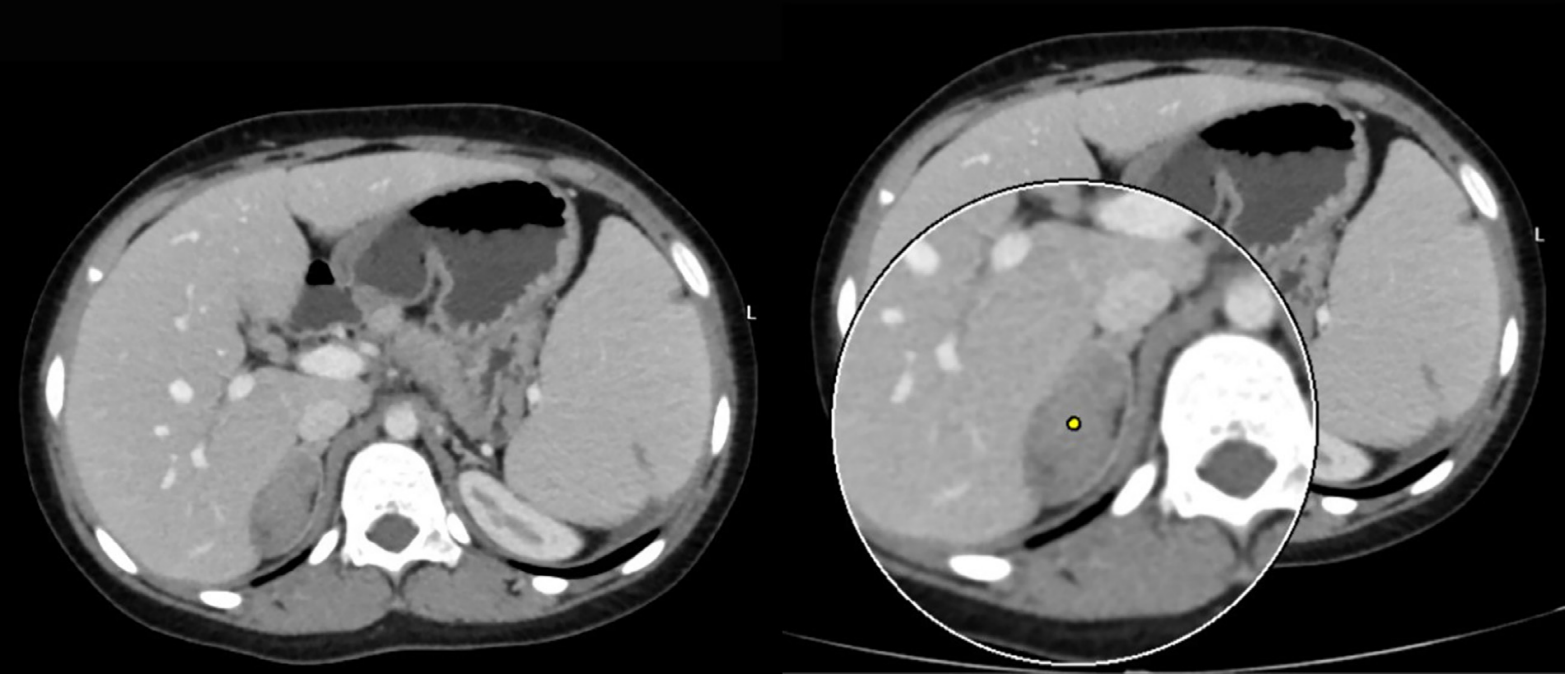
The venous phase showing right adrenal lesion (74HU).

**Fig. 4:**
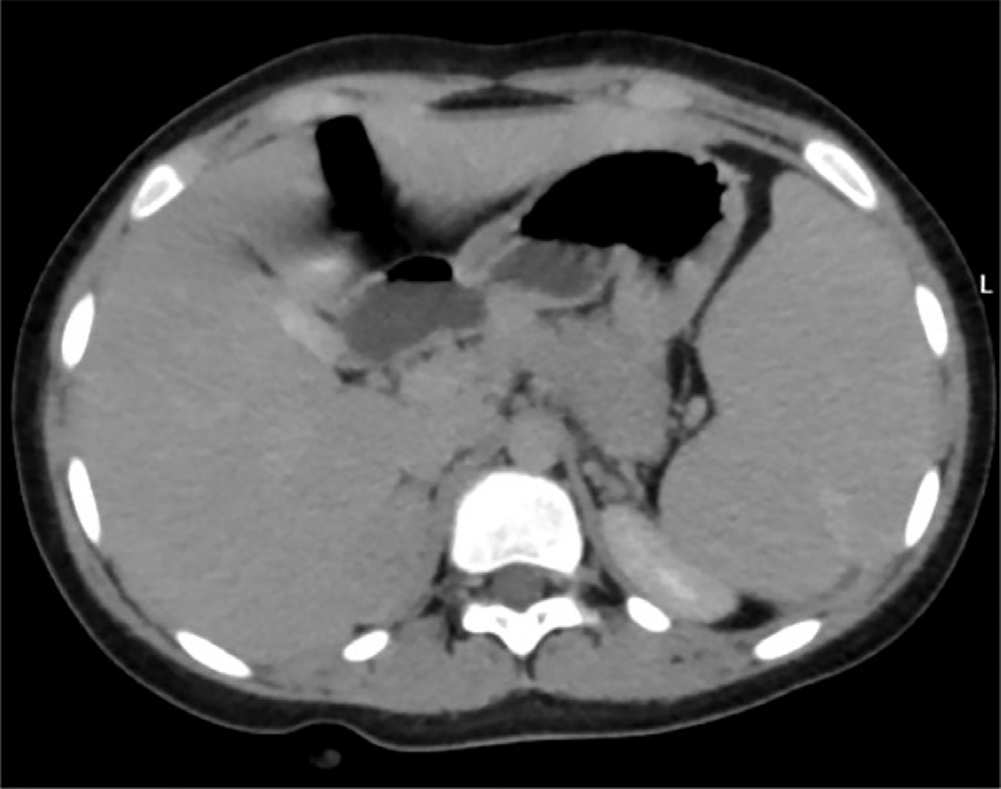
The washout phase showing the right adrenal lesion (69HU).

Blood investigations revealed a hemoglobin level of 10.3 g/dl (Ref. range 11.5-16.5 g/dl), Sodium 140 mmol/L (Ref. range 135 –145 mmol/L), Potassium 4.39 mmol/L (Ref. range 3.5-5.1 mmol/L), Chloride 100 (Ref. range 96-106 mmol/L), ionizing calcium 1.22 mmol/L (Ref. range 1.10-1.40 mmol/L), lactate dehydrogenase of 631 U/L (Ref. range 240-480 U/L), with total bilirubin and direct bilirubin of 3.7 mg/dl (Ref. range 0.1-1.2mg/dl), and 1.47 mg/dl (Ref. range 0-0.3 mg/dl) respectively. Urine volume was 1.2 L/day (Ref. range 0.8-2.0 L/day), metanephrine 178.6 µg/day (Ref. range <350µg/day), serum aldosterone 41.9 pg/ml (13.3-233.5 pg/ml), serum renin 21.1 pg/ml (Ref. range 2.79-61.83 pg/mL), aldosterone/renin ratio 1.98 (Ref. range 0.52-37.83), the 24 hour nor-epinephrine 90.7 nmol/day (Ref. range <535 nmol/day). The blood urea nitrogen and creatinine were within the reference range as well. An abdominal ultrasound scan showed a lobulated outline soft tissue mass lesion at the right suprarenal region, which measured 44 × 30mm with no calcification. In addition, chronic calculous cholecystitis and moderate splenomegaly were detected as well. An abdominal computed tomography (CT) scan showed a right adrenal lesion 48 Hounsfield units (HU) with measurements of 4.1 × 2.2 × 1.5 cm, no fat or calcific content, and normal surrounding tissues. There was no pressure effect, and it showed contact with the surface of the liver without invasion (Fig. 1). After taking contrast as per the standard protocol for adrenal lesions. Which included native, arterial, venous (one minute after intravenous contrast administration), and delayed scans (fifteen minutes after administration). The lesion did not contain fatty tissue. The contrast enhancement measurements of the adrenal glands were 61 HU in the arterial phase (Fig. 2), 74 HU in the venous phase (Fig. 3), and 69 HU in the washout phase (Fig. 4). This results in approximately 40% absolute washout, leading to a diagnosis of an indeterminate lesion. In addition, the CT scan showed an enlarged spleen of 15 cm with a homogenous texture.

### Therapeutic intervention

**Fig. 5:**
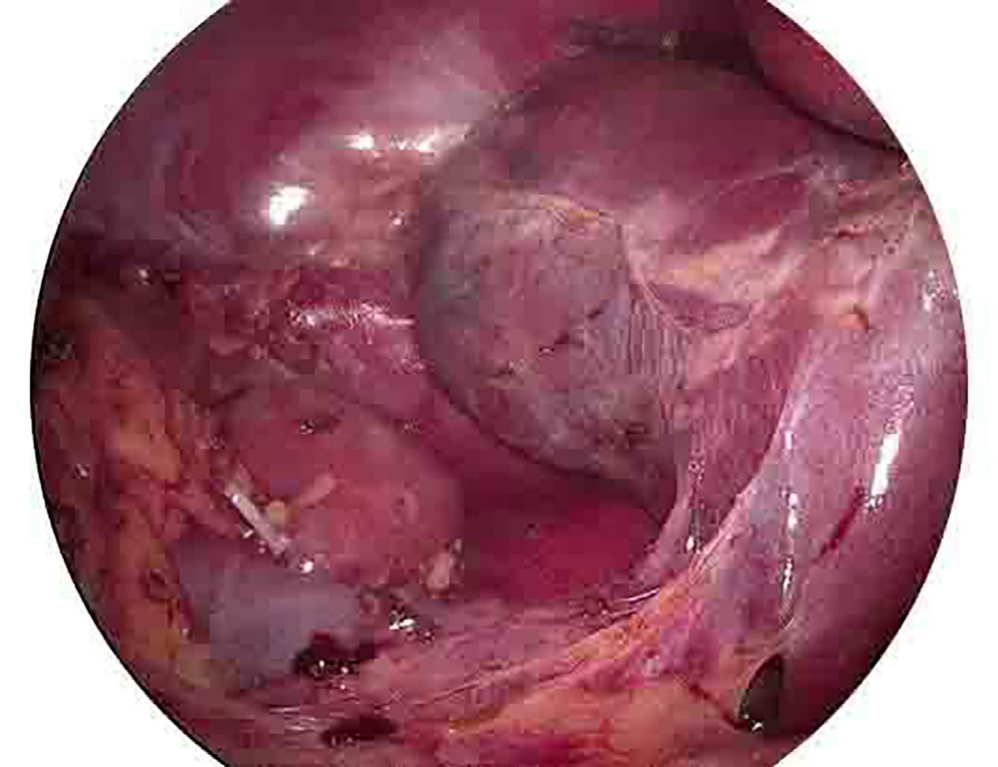
Laparoscopic view after removal of the right adrenal gland.

**Fig. 6:**
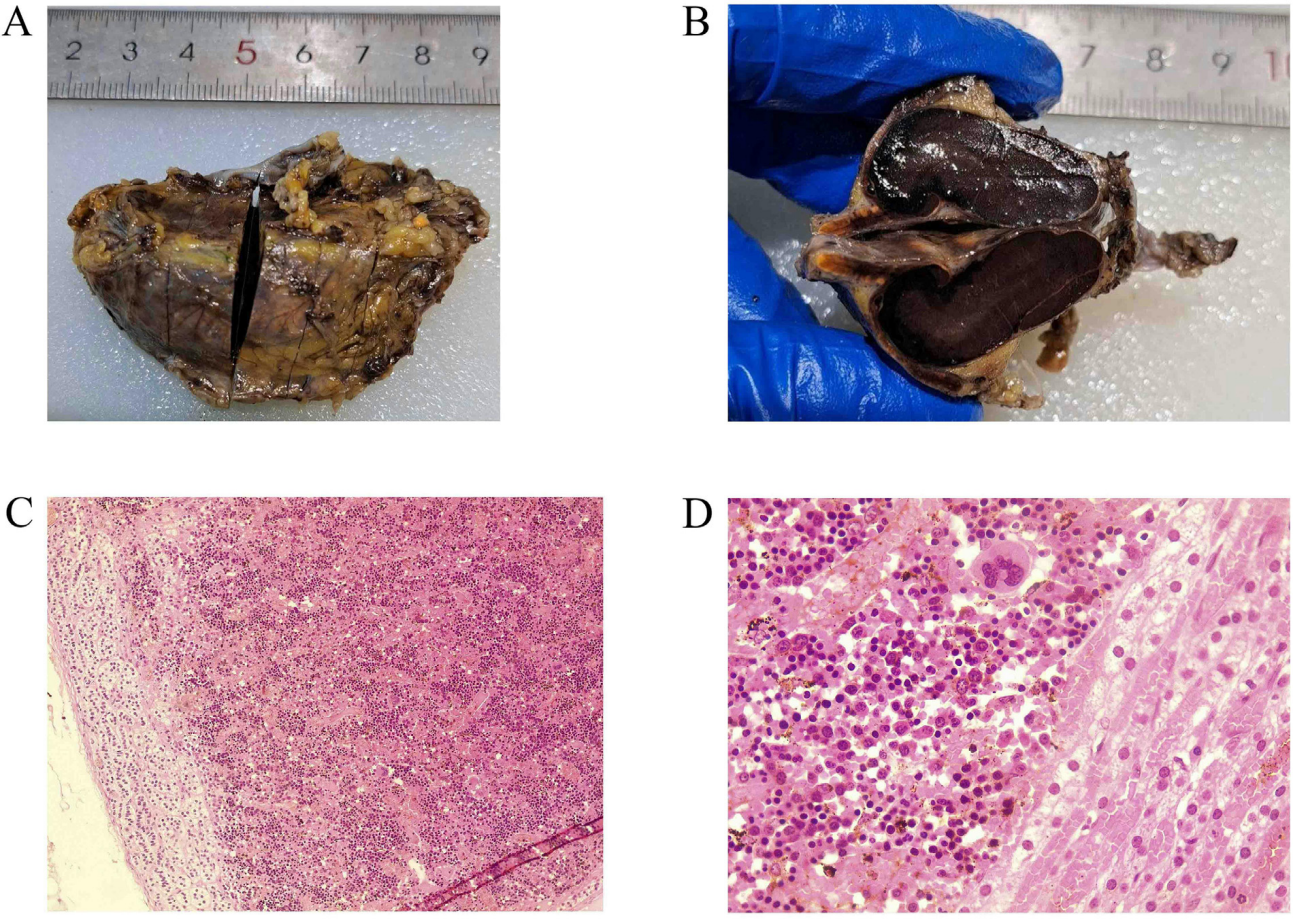
(A) Right adrenal mass excision. (B) Bisection of the mass demonstrates the orange adrenal gland on the left with an attached dark-brown hemorrhagic mass with no yellowish fatty area. (C) the adrenal gland on the left, with nearby mass consisting of hematopoiesis (H&E x100). (D) compressed adrenal tissue on the right, with the EMH showing hematopoietic precursors demonstrating megakaryocytes, admixed myeloid precursors and erythroid precursors (H&E x400).

The decision to surgically remove the adrenal mass was made due to inconsistent radiological features with a typical benign adenoma, coupled with the large size of the tumor. Under general anesthesia, in the supine position, through 4 ports, a laparoscopic cholecystectomy was performed. Afterward, in the left lateral position, a right laparoscopic adrenalectomy was performed. The total duration of the operation was two and a half hours (Fig. 5). Gross histopathologic examination showed the mass demonstrating the orange adrenal gland with attached dark-brown hemorrhagic cut section mass having no yellowish fatty area, with the microscopic examination revealing hematopoietic precursors demonstrating megakaryocytes, admixed myeloid precursors and erythroid precursors with no fatty tissue in spite of extensive sampling (Fig. 6).

### Follow-up and outcome

The patient’s postoperative period was uneventful, with the only complaint of pain at the site of surgery. An abdominal ultrasound showed no other sign of EMH or recurrence of the mass.

## Discussion

Extramedullary hematopoiesis (EMH) is recognized as a compensatory process that arises during disrupted or insufficient hematopoiesis within the bone marrow to meet the body’s physiological demands [15,32]. The spleen and liver are widely recognized areas of EMH as they can synthesize fetal hemoglobin. Moreover, it has been documented in various other locations, such as the lungs, gastrointestinal tract, urinary tract, adrenal glands, prostate, peritoneum, skin, breast, central nervous system, and paravertebral areas. The adrenal glands are one of the rarest sites for such a condition, with only several case reports in the literature (Table 1). Several hypotheses aim to explain how EMH could occur in such a wide range of anatomical areas. Some scholars propose that multipotent stem cells disseminate via the bloodstream, subsequently infiltrating various tissues and organs. In addition, the adrenal gland may possess hematopoietic potential during fetal development, and EMH might arise from the primitive cell remnants, especially during increased demand for erythropoiesis. Another hypothesis is that it could potentially occur due to the displacement of bone marrow cells from adjacent bones, especially in cases involving bone erosions or fractures [14].

**Table 1:** Review of case studies detailing patient demographics, clinical findings, management strategies, and outcomes related to adrenal masses.

References	Study design	No. of case	Country	Age (years)	Sex	Symptom	Physical exam findings	Past surgical history	HGB (mg/dl)	Other significant investigation results	Location	Size (cm)	Management	Biopsy	Associated diseases	Follow-up (months)	Recurrence
King et al. [6]	*	1	USA	66	F	Incidental	-	-	-	-	Bilateral R L	2.5 × 4 -	Conservative	Right adrenal FNA: 4 samples detected EMH, postmortem examination: EMH in both adrenals	Angiogenic myeloid metaplasia, myelofibrosis	Died 5 weeks after biopsy due to bleeding from gastric ulcer	No
Papavasilou et al. [7]	^⁎⁎^	2	Greece	24	M	-	-	-	8.2	-	R	-	Surgical adrenalectomy	-	Thalassemia	1-6 years	No
				41	F	Incidental	-	-	10.7	-	R	-	-	Hematopo- ietic and fat	myelofibrosis		No
Lam et al. [8]	*	1	China	31	M	Right lower costal pain	-	Splenec- tomy	8	High ferritin 2009 mcg/l Tc-99 m-labeled colloid showed increased adrenal uptake bilaterally SPECT scan increased bilateral adrenal uptake	bilateral	-	Conservative	-	Beta- Thalassemia intermedia	-	-
Chuang et al. [9]	*	1	Taiwan	27	F	Palpable mass	Palpable mass	Cholecys- tectomy, splenectomy	-	-	R	7.5 × 5.8	Surgical exploration but without resection after biopsy	EMH	Beta- thalassemia	-	No
Calhoun et al. [10]	*	1	USA	9	M	Jaundice	-	-	-	-	R	5.5 × 5 × 2	Partial adrenalectomy, splenectomy, cholecystectomy	EMH	spherocy- tosis	4 days	-
Arkadop- oulos et al. [11]	*	1	Greece	75	F	Incidental	-	-	-	-	L	8 × 6 × 4	Adrenalectomy, radical modified mastectomy	Adrenal cavernous hemangioma + EMH	Breast cancer	-	-
Porcaro et al. [12]	*	1	Italy	10	F	Incidental	-	-	10	-	R	5	Adrenalectomy	EMH	Beta- thalassemia	72 months, died of infection and heart failure	-
Lau et al. [13]	*	1	China	43	F	Right upper abdominal pain, pallor, jaundice	-	-	-	-	R	7.5	Adrenalectomy	EMH	Hemoglobin H constant spring disease	-	-
Keikhaei et al. [14]	*	1	Iran	26	M	Incidental	Pallor, jaundice, Hepatosplemogaly	-	7.2	Ferritin 2600 ng/ml	R	7.7 × 7.3 × 5.8	Conservative	FNA: EMH	Thalass- emia major	-	N/A
Banerji et al. [15]	*	1	India	40	M	Upper abdominal pain, weight loss, anorexia	Pallor, splenomegaly	-	-	Ferritin 703 ng/ml	R	9 × 8	Adrenalectomy	EMH	Delta-beta thalassemia	6 months	No
Al-Thani et al. [16]	*	1	Qatar	48	F	Right hypochondrial pain + vomiting	Large palpable firm mass	-	6-10	-	R	16 × 11 × 10	Adrenalectomy	Adrenal myelolipoma + EMH	Beta-Thalasemia trait + microcytic iron defeceincy anemia	1 week	No
Nigam et al. [17]	^⁎⁎^	5	India	24	M	Right flank pain	No significant finding	No	14.5	No	L	4.5 × 3.5	Adrenalectomy	EMH	No	-	-
				48	F	Abdominal pain	No significant finding	No	12.6	No	R	10 × 6.5 × 5.5	Adrenalectomy	EMH	No	-	-
				27	F	Vomiting, diarrhea, weight loss	Mild pallor	No	10.4	No	L	24 × 15.5 × 12.5	Adrenalectomy	EMH	No	-	-
				55	M	Incidental	No significant finding	No	13.8	Platelet count: 1.32 × 10^6/μL	R	6.5 × 5.5 × 4.5	Adrenalectomy	EMH	No	-	-
				38	F	Intermittent abdominal pain	No significant finding	No	11.0	Platelet count: 1.29 × 10^6/μL	R	16 × 8.5 × 6	Adrenalectomy	EMH	No	-	-
Tanner et al. [18]	*	1	UK	30	M	Incidental	No significant finding	No	10.5	Bilirubin: 62 μmol/L	R	4	Adrenalectomy + cholecystectomy	EMH	Beta- Thalassemia	-	-
Al-Diab et al. [19]	*	1	Iraq	19	M	Incidental	No significant finding	Splenectomy	6-9	-	R	7.2	Adrenalectomy	EMH	Beta-Thalassemia major	-	-
Kurian et al. [20]	*	1	India	36	M	Incidental	No significant finding	Splenectomy	-	-	Bilateral	7	Conservative	CT-guided biopsy: EMH	Beta-Thalassemia intermedia	-	-
Zachary et al. [21]	*	1	USA	37	-	Incidental	-	Cholecyst- ectomy	-	-	R	5.3 × 3.5	adrenalectomy	Adrenal adenoma with osseus metaplasia and EMH	No	4 months	No
Montalvo-Hernández et al. [22]	*	1	India	55	F	Incidental	-	-	15.8	-	L	3.2 × 2.4	Adrenalectomy	EMH	No	-	-
Kolev et al. [23]	*	1	Bulga- ria	50	M	Incidental	No significant finding	-	12.2	-	R	8 × 8	Radical nephron-adrenalectomy	EMH + mature adipose tissue with extensive hemorrhages	No	-	-
Wong et al. [24]	*	1	Malaysia	44	F	Incidental	Jaundice, large palpable firm mass, and huge splenomegaly	No	7.6–9.9	Bilirubin: 54 μmol/L	R	14.8 × 11 × 9.8	Conservative (hypertransfusion, iron chelation, hydroxyurea) with resultant decrease in size of mass	-	Thalass- emia intermedia	-	-
Korkmaz et al. [25]	*	1	Turkey	33	M	Incidental	-	Splene- ctomy	10.7	FLT-PET scan showed increased uptake in both adrenal glands	Bilateral	Right: 5.0 × 3.5 Left: 4.5 × 3.3	Conservative	Biopsy: EMH	Thalass- emia major	-	-
Georgiu et al. [26]	*	1	Cyprus	40	F	Abdominal pain	Tenderness on right flank, splenomegaly	-	8.7-10.4	Bilirubin 3.0-4.8 mg/dl	R	5.8 × 4.2 × 4.6	Adrenalectomy	EMH + occasional adipocytes	Beta- Thalassemia	2 years	No
Julson et al. [27]	*	1	USA	16	F	Incidental	-	-	-	-	R	5.2 × 4.3 × 4.8	1 year of conservative management but then due to increase in size adrenalectomy was ultimately done	EMH	Anti-Diego antibody and congenital dyserythropoietic anemia	-	-
Ajayi et al. [3]	*	1	USA	21	M	Thoracic pain	No significant finding	-	7.8	Bilirubin: 2 Reticulocyte: 7.8% LDH: 874	R	4.7 × 2.7	Conservative	-	Homozy- gous sickle cell disease	2 months	No change in size
Jana et al. [28]	*	1	India	48	M	Flank pain	-	Splenec- tomy	-	-	R	12 × 12	Adrenalectomy	Adrenal myelolipoma +EMH + hemorrhage	Hereditary spherocytosis	18 months	No
Meletli et al. [29]	*	1	Turkey	67	F	-	-	-	-	-	R	-	Adrenalectomy	Cortisol secreting adrenocortical adenoma containing EMH	Myelodysp- lastic syndrome	-	-
Gupta et al. [4]	*	1	India	41	F	Right hypochondrial pain	Hepatosple- nomegaly + palpable lumbar mass	-	1.5	LDH: 3200 Platelet: 7740	R	15 × 11.5 × 7.5	Adrenale- ctomy + splenectomy	EMH	Sickle cell trait	-	-

Abbreviations: M, male; F, female; EMH, extramedullary hematopoiesis; FLT-PET, fluorothymidine positron emission tomography; LDH, lactate dehydrogenase; HGB, hemoglobin; R, right; L, left.

*Case report.

^⁎⁎^Case series.

Georgiu et al. presented a 40-year-old female with beta-thalassemia who complained of abdominal pain. After investigation with ultrasound and CT scan, it revealed a right adrenal mass with a size of 5.8 × 4.6 × 4.2 cm. It is worth mentioning that the patient's hemoglobin level ranged between 8.7-10.4 mg/dl. The patient underwent an adrenalectomy with histopathological examination showing EMH with occasional adipose tissue [24]. This was in accordance with the present patient in which the mass was located on the right side, and the EMH of the adrenal mass diagnosis was made only after adrenalectomy and histopathological examination were conducted. It is worth noting that 75% of EMH in the adrenals in the literature, including the present case, were located on the right side, in comparison to the left side or bilateral involvement of both glands, with each instituting 12.5%. This disparity between the right and left adrenal gland involvement needs more data and research to confirm it. Still, perhaps this observation will give clues regarding the underlying pathophysiology of EMH. For instance, the right adrenal gland might be a more favorable site, providing better anatomical access for dislodged hematopoietic cells from the bone marrow to settle, or perhaps the right adrenal gland might harbor the hypothesized dormant hematopoietic stem cell remnants better compared to the left side. Another notable observation from reviewing the literature was that 72% of the cases were associated with hematologic disorders, which is in accordance with the current patient who had hereditary spherocytosis. However, what sets this case apart from the other reported cases in the literature is that EMH in the adrenal glands mostly occurs in adult patients, with only 3 reported cases being in the pediatric age group. One of them was reported by Calhoun et al., involving a 9-year-old female patient with spherocytosis who had a 5.5 × 5 × 2 cm right adrenal mass and underwent partial adrenalectomy [10]. Another interesting case was showcased by Julson et al., featuring a 16-year-old female with a right-sided adrenal mass of 5.2 × 4.3 × 4.8 cm in size. The patient had anti-Diego antibody and congenital dyserythropoietic anemia. They opted for a conservative approach for one year; however, the mass kept increasing in size. Ultimately, an adrenalectomy was done [27]. The third reported case in a pediatric patient was by Porcaro et al., encompassing a 10-year-old female patient with Beta thalassemia of unspecified subtype who had a right adrenal mass, and an adrenalectomy was performed. Unfortunately, 72 months later, the patient died due to infection and heart failure [12]. The current patient represents the fourth reported case of EMH in the adrenal gland of a pediatric patient.

The symptoms and manifestations of EMH in the adrenal glands can vary and are influenced by the size and location of the hematopoietic tissue. For some individuals, the condition may be asymptomatic and only detected incidentally during imaging studies conducted for unrelated reasons. Conversely, in other instances, EMH can manifest as a palpable abdominal mass or produce symptoms attributable to its impact on adjacent structures. For instance, Keikhaei et al. reported a 26-year-old man with Beta thalassemia major with the incidental diagnosis of a 7.7 × 7.3 × 5.8 cm adrenal mass on routine ultrasonography. However, Nigam et al. highlighted a case that presented with intermittent abdominal pain, which was later found to have a 16 × 8.5 × 6 cm adrenal mass and underwent surgical resection to relieve the symptoms and was found to have EMH on histopathological examination [14,17]. The current patient presented with dull right hypochondrial pain, and on further investigation, the mass was found, and surgery was recommended due to the risk of malignancy.

Thorough imaging studies and adrenal hormonal assessments are crucial to exclude cancer and subclinical hypersecretory conditions from the adrenal glands. Advanced imaging modalities, including CT scans and magnetic resonance imaging (MRI), are employed in evaluating patients presenting with retroperitoneal masses. These diagnostic tools are paramount in accurately determining the tumor's dimensions and precise anatomical location. Furthermore, they provide detailed visualization of the tumor's spatial relationship with surrounding structures, a critical factor in the surgical management of these cases. This meticulous imaging assessment is particularly vital given the high likelihood of malignancy associated with retroperitoneal tumors. Over the past 2 decades, advancements in radiological imaging have led to a higher detection rate of clinically asymptomatic benign adrenal tumors, commonly referred to as adrenal incidentalomas. These incidental findings represent a proportion estimated to range between 1% and 5% of all abdominal CT scans conducted for various medical purposes [11,[33], [34], [35]]. Asymptomatic patients with adrenal lesions usually undergo conservative management, whereas symptomatic patients might require treatment [16]. In the review of diagnostic imaging for the cases (Table 2), a detailed description was not provided for all cases; some only mentioned the presence of an adrenal mass. However, in cases where the mass was described in greater detail, noncontrast CT revealed heterogeneous density in nearly 90% of cases, while the remaining cases were reported as homogeneous. On contrast-enhanced CT, 50% of adrenal masses exhibited heterogeneous enhancement, 20% were homogeneous, 10% were unspecified, and 20% showed no enhancement. The adrenal gland in the current patient showed homogenous enhancement on contrast CT with arterial and venous phases showing 61 HU and 74 HU, respectively. MRI was performed in a minority of cases, with insufficient details to draw definitive conclusions. However, in the case reported by Calhoun et al., MRI showed low signal intensity on T1-weighted images and a bright signal on T2-weighted images. Tanner et al. described focal high T2 signal intensity in the adrenal gland, while Ajayi et al. reported a gradually enhancing retroperitoneal mass [3,10,18]. In addition, ultrasonography findings were variable, with the adrenal mass appearing hypoechoic, hyperechoic, or mixed, often with a well-defined border (Table 2). In the review of the literature, the vast majority of patients have undergone adrenalectomy. This may be due to the fact that EMH of the adrenal glands often goes undiagnosed in cases where the mass is not surgically removed, as such cases may be misclassified as incidentalomas on imaging. This could explain the high rate of surgical management observed for this condition.

**Table 2:** Radiological findings of adrenal hematopoietic lesions.

References	Location	Size (cm)	U/S	CT	MRI
King et al. [6]	Bilateral R L	2.5 × 4 -	-	On nonenhanced CT the right adrenal mass showed mild inhomogeneous density. The left adrenal showed non focal enlargement. The liver showed high density due to hemosiderosis from recurrent blood transfusions.	-
Papavasilou et al. [7]	R	-	-	Adrenal mass	-
	R	-	-	-	-
Lam et al. [8]	Bilateral	-	Bilateral adrenal enlargement, hepatomegaly	-	Bilateral adrenal mass
Chuang et al. [9]	R	7.5 × 5.8	Suprarenal mass	Suprarenal mass	Suprarenal mass
Calhoun et al. [10]	R	5.5 × 5 × 2	5 cm lobulated solid hypoechoic suprarenal mass, splenomegaly, cholelithiasis	Non contrast CT showed solid lobulated suprarenal mas, enlarged spleen	The mass appeared of adrenal origin, and showed low signal intensity on T1, a heterogeneously bright signal on T2, and only slight enhancement after gadolinium administration.
Arkadopoulos et al. [11]	L	8 × 6 × 4	A heterogeneous solid lesion of the left adrenal gland.	Well-defined, heterogeneous, retroperitoneal mass with speckled calcifications that measured 8 cm and was located on the left adrenal gland. After bolus iv injection of contrast medium the tumor showed irregular enhancement.	Tumor with fat component and irregular peripheral enhancement
Porcaro et al. [12]	R	5	Right adrenal gland with a well-defined, hypoechoic, round mass	Non homogenous, hypodense adrenal mass	-
Lau et al. [13]	R	7.5	Hepatosplenomegaly, cholelithiasis, and an oval well-encapsulated solid right suprarenal mass, the mass showed a homogeneous hypoechoic echo pattern. It contained no calcifications or cystic components. No notable increase in vascularity was noted on color doppler interrogation	An enhancing right adrenal mass with focal areas of hypoenhancement. The mass measured 40 and 80 hounsfield units on noncontrast and 60-second postcontrast scans, respectively	-
Keikhaei et al. [14]	R	7.7 × 7.3 × 5.8	Showed huge hepatosplenomegaly and a well-defined right suprarenal solid mass	Adrenal mass	-
Banerji et al. [15]	R	9 × 8	Enlarged adrenal gland	Contrast enhanced CT of the abdomen and pelvis revealed splenomegaly, and a homogenous moderately enhancing lesion in the region of the right adrenal gland, replacing it almost completely.	-
Al-Thani et al. [16]	R	16 × 11 × 10	Well-defined hyperechoic heterogeneous mass in the right upper quadrant. Smooth outline and was associated with minimal vascularity	Computerized tomography scan of the abdomen with oral and intravenous contrast showed a well-defined encapsulated retroperitoneal lesion. It was of mostly fat density with internal areas of soft tissue density in the hepatorenal space. It was seen displacing the right kidney inferiorly with clear and preserved demarcation between the 2 structures. The mass also displaced the inferior vena cava, duodenum and pancreatic head anteromedially. The right adrenal gland was not visualized separately.	Large right-sided retroperitoneal predominantly fatty mass in the location of right adrenal gland. The mass was definitely extra-renal in location pushing the right kidney down. It rose from behind the right aspect of the pancreas, IVC and second/third part of duodenum which expanded along the anterior aspect of the mass and indented over the right liver lobe margin without definite infiltration. The mass demonstrated some soft tissue nodular and linear strand-like areas which showed mild enhancement on postcontrast images.
Nigam et al. [17]	L	4.5 × 3.5	-	-	-
	R	10 × 6.5 × 5.5	-	An enlarged right adrenal gland with presence of fat	-
	L	24 × 15.5 × 12.5	-	A large hypodense left retroperitoneal mass with fat density	-
	R	6.5 × 5.5 × 4.5	Adrenal mass	Well-defined and appeared heterogeneous	-
	R	16 × 8.5 × 6	-	Well defined, heterogeneously nonenhancing lesion in the right suprarenal region	-
Tanner et al. [18]	R	4	Adrenal mass + cholelithiasis	Unenhanced axial CT image showed a homogeneous right adrenal mass. Average attenuation of the mass was 41 HU. Axial arterial phase CT image showed homogeneous enhancement of the adrenal mass. Average attenuation of the mass was 64 HU. Axial arterial phase CT image showed a right paraspinal soft-tissue mass. Axial portal venous phase CT image showed a dependent high-attenuation layer within the gallbladder, consistent with sludge or stone	Coronal T2-weighted showed adrenal mass and splenomegaly. There was a region of focal high T2 signal intensity within the adrenal mass. Axial dual-echo in-phase MR image showed signal loss anteriorly within the mass relative to that seen with opposed phase imaging. Axial dual-echo opposed-phase MR image showed focal signal loss posterolaterally within the right adrenal mass
Al-Diab et al. [19]	R	7.2	Adrenal mass	Adrenal mass	-
Kurian et al. [20]	Bilateral	7	Bilateral adrenal mass	Well-defined, bilateral and nonenhancing adrenal masses	-
Zachary et al. [21]	R	5.3 × 3.5	-	-	-
Montalvo-Hernández et al. [22]	L	3.2 × 2.4	-	Heterogeneous adrenal mass with small calcifications inside the left adrenal gland with a 28 HU in the noncontrast phase, enhancement in the arterial phase, and an absolute washout of 58%	-
Kolev et al. [23]	R	8 × 8	Solid suprarenal mass with heterogenous eco-structure	Heterogenous adrenal lesion with presence of adipose tissue centrally up to 60 HU and denser sections peripherally up to 40 HU, replacing the gland almost completely	-
Wong et al. [24]	R	14.8 × 11 × 9.8	Right liver lobe mass	Adrenal mass + paraspinal mass	-
Korkmaz et al. [25]	Bilateral	Right:5.0 × 3.5 Left:4.5 × 3.3	-	Bilateral adrenal mass	Well-circumscribed, solid mass in the right adrenal gland and solid mass in the left adrenal gland, in out-of-phase sections without significant signal loss.
Georgiu et al. [26]	R	5.8 × 4.2 × 4.6	Adrenal mass + hepatomsplenomegaly	Adrenal mass + hepatosplenomegaly	Prior routine MRI did not detect it
Julson et al. [27]	R	5.2 × 4.3 × 4.8	Adrenal mass	Adrenal mass	Adrenal mass
Ajayi et al. [3]	R	4.7 × 2.7	A mass impressing on the posterior wall of the suprarenal inferior vena cava extending into the lumen	-	MRI with and without contrast revealed a progressively enhancing right retroperitoneal mass
Jana et al. [28]	R	12 × 12	Hepatomegaly, gallstone, and a mixed echogenic mass in the right suprarenal region	Contrast-enhanced CT revealed evidence of well-defined, lobulated outlined, mixed solid, and fat density focus on the right adrenal gland with nearly 30%-40% fat contents seen in the lateral aspect of the focus. Mild enhancement of the solid components was seen. There was no evidence of any microcalcification. The right kidney was pushed inferiorly. The left adrenal gland was unremarkable. Multiple enlarged paravertebral soft tissue density foci were seen along the lower dorsal spine.	-
Meletli et al. [29]	R	-	-	-	Adrenal mass with heterogeneous signal loss
Gupta et al. [4]	R	15 × 11.5 × 7.5	Right suprarenal mass with splenomegaly, a 1.2 cm calculus in the gallbladder, and a 2.4 cm calculus in the right renal pelvis.	A well-defined lobulated lesion with heterogenous postcontrast enhancement with nonenhancing necrotic areas in the right suprarenal region, displacing the kidney inferiorly, liver anteriorly, and portal vein and IVC medially without any vascular invasion + splenomegaly	Adrenal mass + splenomegaly

If there is high clinical suspicion and EMH is included in the differential diagnosis of adrenal masses in cases with a normal adrenal biochemical evaluation, especially in patients with a known hematologic condition that increases their chance of EMH, the diagnosis can be confirmed using image-guided adrenal biopsy, which will aid in avoiding adrenalectomy. Sekar et al. demonstrated this approach in a patient with beta-thalassemia using ultrasound-guided biopsy, while King et al. and Kurian et al. utilized CT-guided biopsy to reach the diagnosis of EMH in the adrenal gland and avoid surgery successfully [6,20,36]. Furthermore, Lam et al. indicated the presence of reticuloendothelial tissue, as revealed by bone marrow scintigraphy using Technetium Tc-99m nanocolloid. This case highlights the application of noninvasive functional imaging techniques in managing adrenal incidentalomas [8]. Another unique diagnostic approach was for a bilateral adrenal mass reported by Korkmaz et al. that showed Positron emission tomography using fluorodeoxyglucose (18F-FDG), revealing slight FDG uptake in both adrenal masses excluding malignancy. Subsequently, Fluorothymidine (18F-FLT) Positron emission tomography was conducted. This revealed peripheral, nonhomogeneous increased FLT uptake in both adrenal glands, with a maximum standardized uptake value of 8.2 in the left and 6.7 in the right. Adrenal biopsy from the periphery of the left adrenal gland, where intense FLT uptake was observed, confirmed EMH [25].

Specific interventions may not be necessary unless EMH in patients manifests with symptoms. Treatment strategies for managing EMH depend on its location and the presence of symptoms and may involve surgical procedures, radiation therapy, blood transfusions, hydroxyurea administration, or a combination thereof. Radiotherapy alone has shown promising outcomes in treating spinal cord compression caused by EMH, with reported excellent results. In addition to stimulating hemoglobin F synthesis, hydroxyurea may actively contribute to the inactivation and reduction in size of EMH by enhancing erythropoiesis efficiency in individuals with β-thalassemia [14]. Adrenalectomy can be pursued as well when the mass is large and causing symptoms or when malignancy cannot be excluded.

## Conclusion

Patients with hereditary spherocytosis may present with an adrenal mass due to EMH, even in pediatric age groups. In patients with known hematologic diseases, preoperative diagnosis through functional imaging or biopsy may prevent the need for surgical intervention.

## Availability of data and material

All data and materials are kept by the first and corresponding authors.

## Patient consent

Consent has been taken from the patients and the family of the patients.
